# The association between vitamin D supplementation and the long-term prognosis of differentiated thyroid cancer patients: a retrospective observational cohort study with propensity score matching

**DOI:** 10.3389/fendo.2023.1163671

**Published:** 2023-06-13

**Authors:** Jong-hyuk Ahn, Hoonsung Choi, Su-jin Kim, Sun Wook Cho, Kyu Eun Lee, Do Joon Park, Young Joo Park

**Affiliations:** ^1^ Department of Surgery, Chungbuk National University Hospital, Cheongju, Republic of Korea; ^2^ Department of Surgery, lnha University College of Medicine, Incheon, Republic of Korea; ^3^ Department of Internal Medicine, Chung-Ang University College of Medicine, Seoul, Republic of Korea; ^4^ Department of Surgery, Seoul National University Hospital & Seoul National University College of Medicine, Seoul, Republic of Korea; ^5^ Cancer Research Institute, Seoul National University College of Medicine, Seoul, Republic of Korea; ^6^ Department of Internal Medicine, Seoul National University Hospital & Seoul National University College of Medicine, Seoul, Republic of Korea; ^7^ Department of Internal Medicine and Genomic Medicine Institute, Medical Research Center, Seoul National University College of Medicine, Seoul, Republic of Korea; ^8^ Department of Molecular Medicine and Biopharmaceutical Sciences, Graduate School of Convergence Science and Technology, Seoul National University, Seoul, Republic of Korea

**Keywords:** vitamin D, thyroid carcinoma, propensity score, mortality, cause of death

## Abstract

**Objective:**

Benefits of vitamin D in various cancers have been reported, but its effects on differentiated thyroid cancer (DTC) have not been established. We aimed to analyze the effect of vitamin D supplementation on the prognosis of DTC.

**Methods:**

A retrospective observational cohort study was conducted on 9,739 DTC patients who underwent thyroidectomy from January 1997 to December 2016. Mortality was classified as all-cause, cancer-related, or thyroid cancer-related. Patients were divided into the “VD group” (supplemented with vitamin D) and the “control group” (without vitamin D supplementation). Propensity score matching was performed in a 1:1 ratio according to age, sex, tumor size, extrathyroidal extension (ETE), and lymph node metastasis (LNM) status, and 3,238 patients were assigned to each group. Kaplan-Meier curves, log-rank test and Cox proportional hazards regression analysis were performed.

**Results:**

The follow-up period was 10.7 ± 4.2 years. Clinicopathological variables between two groups were similar except for all-cause (*p*<0.001) and total cancer death (*p*=0.001). From the Kaplan−Meier curve and log-rank test, “VD group” had significantly favorable all-cause (*p*<0.001) and total cancer mortality (*p*=0.003), but similar thyroid cancer mortality (*p*=0.23). In Cox regression, vitamin D intake reduced the risk of all-cause (hazard ratio [HR], 0.617, *p*=0.001) and total cancer mortality (HR, 0.668, *p*=0.016) but had no effect on thyroid cancer mortality.

**Discussion/conclusion:**

Vitamin D supplementation was positively associated with all-cause and total cancer mortality in DTC and might be a modifiable prognostic factor for improved survival. Further research will be needed to clarify the effect of vitamin D supplementation on DTC.

## Introduction

The incidence of thyroid cancer has increased significantly worldwide, and thyroid cancer accounts for 3% of all cancers worldwide (1). Most of the diagnosed thyroid cancers are differentiated thyroid cancer (DTC), including papillary thyroid cancer (80-85%) and follicular thyroid cancer (10-15%), and the prognosis is generally excellent (2, 3). However, recurrence (10-15%) or distant metastasis (5%) may occur in DTC patients even after they have received appropriate treatment such as surgery or radioactive iodine treatment (2).

It has been reported that patients with poor prognostic factors have a higher risk of recurrence and death (4). There are several established prognostic factors in thyroid cancer, such as age, sex, tumor histopathology, tumor size, lymphovascular invasion, extrathyroidal extension (ETE), lymph node metastasis (LNM), distant metastasis, *BRAF* mutation, and *TERT* promoter mutation (3, 5). Since these prognostic factors cannot be modified, many studies are being conducted to explore modifiable prognostic factors in thyroid cancer patients (6, 7).

Vitamin D plays an important role in the body’s calcium homeostasis (8). Vitamin D also exerts action in the tumor microenvironment, which is the environment surrounding a tumor and can influence the growth of cancer cells, by inhibiting proliferation, inflammation, invasion, metastasis and angiogenesis and by inducing apoptosis and cell differentiation (3, 8, 9). Several studies have shown that vitamin D supplementation affords good results in the incidence and prognosis of cancer, particularly breast, prostate, and colorectal cancer (10, 11). However, the effects of vitamin D supplementation on the prognosis of cancer are still controversial. One meta-analysis reported that vitamin D intake significantly reduced total cancer mortality by 13%, whereas another meta-analysis reported that vitamin D intake had no effect on reduction in total cancer mortality (12, 13).

Only a few studies have investigated the role of vitamin D in the aggressiveness and prognosis of thyroid cancer. Some studies reported that thyroid cancer patients had lower vitamin D levels, and lower vitamin D levels were associated with advanced thyroid cancer, characterized variably by higher T stage, ETE, and LNM (14, 15). Other studies reported that vitamin D levels had no association with the risk of incidence, advanced stage, or prognostic features of thyroid cancer (16, 17). There have been no studies analyzing the effect of vitamin D supplementation on mortality in thyroid cancer patients. The purpose of this study was to elucidate the potential role of vitamin D supplementation as a modifiable risk factor in the prognosis of thyroid cancer. We hypothesized that vitamin D supplementation would have a positive effect on mortality in thyroid cancer patients.

## Methods

**Figure 1:**
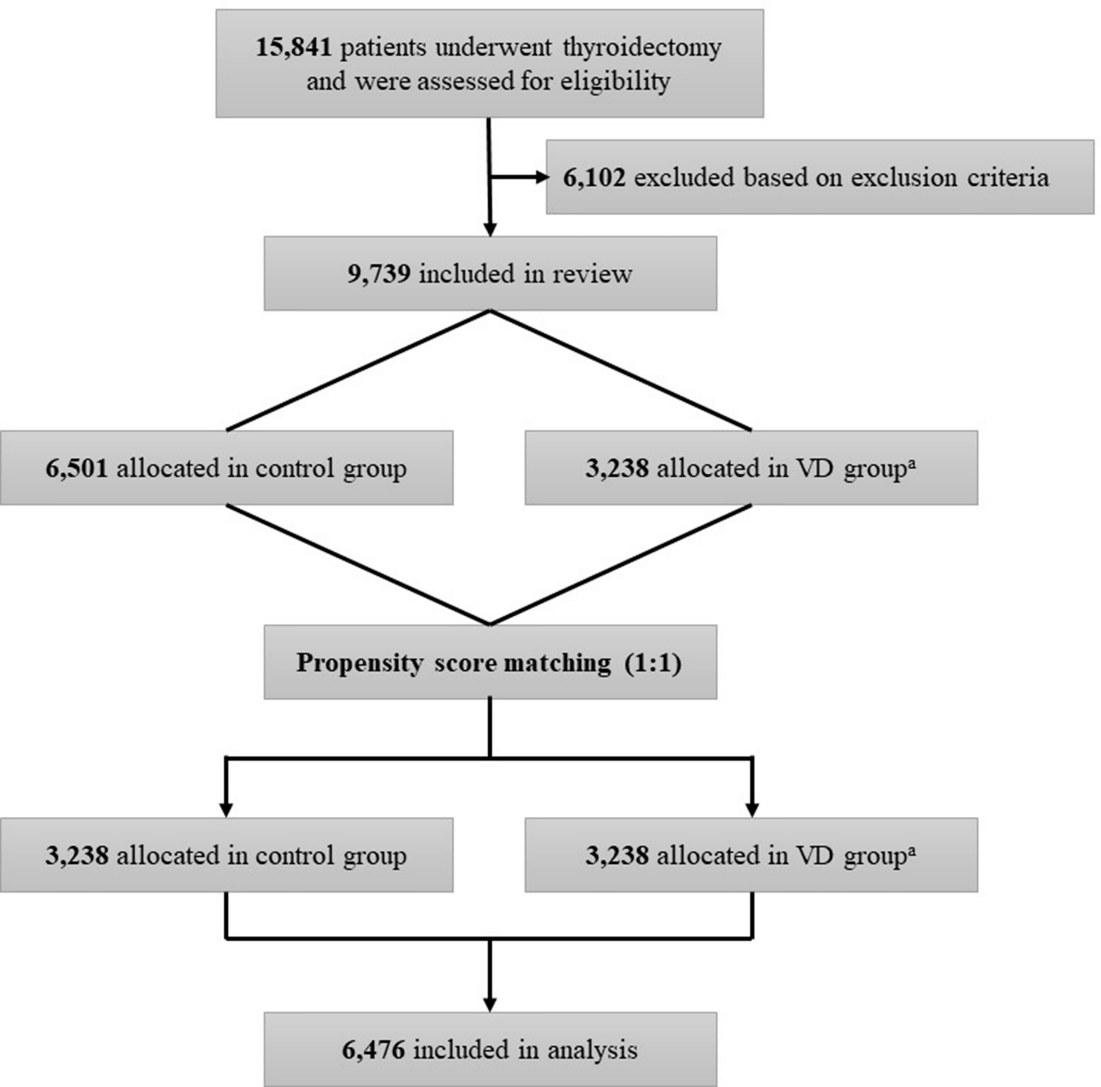
Flow chart of the study. ^a^VD group is the case group with oral vitamin D supplementation for at least 6 months.

We conducted a retrospective observational cohort study. The flow diagram of this study is described in **Figure 1**. The cohort included patients aged 19 to 70 years who underwent thyroidectomy for DTC, including papillary thyroid cancer, follicular thyroid cancer, and Hürthle cell (oncocytic) thyroid cancer, at Seoul National University Hospital between January 1, 1997, and December 31, 2016. The following patients were excluded from this study: 1) patients with unavailable information on vitamin D intake; 2) patients with vitamin D supplementation after surgery for less than six months; 3) patients with a history of other cancers at the time of diagnosis of thyroid cancer; 4) patients with diseases or conditions that cause hypercalcemia or hypercalciuria (e.g., multiple myeloma, bone metastases, or malignant bone disease); 5) patients with intestinal diseases that could affect serum vitamin D levels (celiac disease, small intestine resorption disease, and a history of small bowel resection); 6) patients taking medications that could affect serum calcium or vitamin D levels; and 7) patients unable to have clinical data collected.

A total of 9,739 patients were eligible for this study. Clinicopathologic and survival status data up to December 31, 2019, were collected. Clinicopathological information, including age at thyroid surgery, sex, operation extent, tumor size, ETE, LNM status, and TNM stage, was collected from electric medical records. Operation extent is grouped into ‘TT’ and ‘less than TT’. ‘TT’ includes total thyroidectomy and near total thyroidectomy. ‘Less than TT’ was all surgeries except ‘TT’. ETE was confirmed as positive or negative by the final pathological findings, and ETE included microscopic and gross ETE. Data on the survival status of patients, duration of survival, and cause of death were collected from the registered data of the Korea Statistics. The causes of death were classified into the following three categories: 1) all-cause mortality, defined as patient death for any reason; 2) total cancer mortality, defined as patient death related to all malignancies; and 3) thyroid cancer mortality, defined as patient death related to thyroid cancer.

Vitamin D supplementation was defined as taking active or inactive vitamin D orally for any cause. Patients were divided into two groups according to their vitamin D supplementation status: 1) a control group of patients who did not receive vitamin D supplementation after thyroid surgery and 2) a “VD group” of patients who received supplementation with vitamin D for at least 6 months after thyroidectomy.

The consent of participants was waived due to the nature of the retrospective study. The study was approved by the Institutional Review Board (No. 2012-081-1181). This study followed the Strengthening the Reporting of Observational Studies in Epidemiology (STROBE) Statement reporting guideline for observational studies (18).

### Statistical analysis

To correct the bias of confounding factors, propensity score matching (PSM) using nearest neighbor matching was introduced. Patients in both groups were matched at a 1:1 ratio based on age at the time of thyroid surgery, sex, tumor size, ETE, and LNM status. Considering collinearity, TNM stage was not included as a covariate, because age, tumor size, LNM, and ETE were included in TNM stage. Continuous variables were analyzed using Student’s t test, and categorical variables were analyzed using the chi-square test. All-cause, total cancer, and thyroid cancer mortality in the control and VD groups were compared using the Kaplan−Meier method and log-rank test. Cox proportional hazards regression analysis was used to analyze independent prognostic factors for all-cause, total cancer, and thyroid cancer mortality in thyroid cancer patients. Statistical significance was set at *p* < 0.05. All statistical procedures were performed using R software (R ver. 4.1.3; R Foundation for Statistical Computing).

## Results

### Baseline characteristics

The baseline characteristics of the original unmatched cohort and PSM-adjusted cohort are presented in **Table 1**. A total of 9,739 patients were included in the present study. There were 6,501 (66.8%) patients in the control group and 3,238 (33.2%) in the VD group. Compared to the control group, the VD group in the total cohort demonstrated associations with age at operation (older age), operation extent, ETE, LNM status, and TNM stage (all *p*<0.001). In the control group, higher proportions of patients with all-cause, total cancer, and thyroid cancer mortality were revealed (*p*<0.001, *p*=0.002, and *p*=0.388, respectively). A total of 276 and 77 participants in the control and VD groups, respectively, died during the study period, and the causes of death for each group are summarized in **Supplementary Table 1**. No significant differences in the causes of death by organ system were observed between the control and VD groups. **Supplementary Table 2** summarizes mortality rates based on the extent of the operation and TNM stage in both cohorts. The ‘Less than TT’ group comprised 940 patients, while the ‘TT’ group had 8,671 patients. There were no significant differences in the mortality rates of all-cause, total cancer, and thyroid cancer according to operation extent (all p>0.05). The all-cause mortality was 249 (2.9%) in TNM stage I, 96 (9.8%) in TNM stage II, 5 (13.2%) in TNM stage III, and 3 (60.0%) in TNM stage IV. For all cancer mortality rates, the rates in TNM stage I, II III, and IV were 1.8% (n=158), 7.5% (n=74), 13.2% (n=5), and 60.0% (n=3), respectively. In terms of thyroid cancer mortality rates, they were 0.4% (n=39), 2.8% (n=27), 7.9% (n=3), and 60.0% (n=3) in stages I to IV, respectively.

**Table 1:** Baseline characteristics of original unmatched cohort and propensity score matching-adjusted cohort.

	Original unmatched cohort			PSM-adjusted cohort^a^		
	Control group (n = 6,501)	VD group^b^ (n = 3,238)	*p*-value	Control group (n = 3,238)	VD group^b^ (n = 3,238)	*p*-value
Age at operation (years)	46.2 ± 11.5	48.6 ± 10.8	<0.001	48.5 ± 11.1	48.6 ± 10.8	0.762
Sex			<0.001			0.389
Female	5297 (81.5%)	2896 (89.4%)		2918 (90.1%)	2896 (89.4%)	
Male	1204 (18.5%)	342 (10.6%)		320 (9.9%)	342 (10.6%)	
OP extent^c^	(n=6,375)	(n=3,236)	<0.001	(n=3,169)	(n=3,236)	<0.001
TT^d^	5579 (85.8%)	3092 (95.5%)		2806 (88.5%)	3092 (95.6%)	
Less than TT^d^	796 (12.2%)	144 (4.4%)		363 (11.5%)	144 (4.4%)	
Tumor size (cm)	1.2 ± 1.0	1.2 ± 0.9	0.287	1.2 ± 1.0	1.2 ± 0.9	0.613
ETE^e^			<0.001			0.819
No	2962 (45.6%)	1275 (39.4%)		1285 (39.7%)	1275 (39.4%)	
Yes	3539 (54.4%)	1963 (60.6%)		1953 (60.3%)	1963 (60.6%)	
LNM status^f^			<0.001			0.473
No LNM^f^	4019 (61.8%)	1826 (56.4%)		1847 (57.0%)	1826 (56.4%)	
Central LNM^f^	1365 (21.0%)	830 (25.6%)		789 (24.4%)	830 (25.6%)	
Lateral LNM^f^	1117 (17.2%)	582 (18.0%)		602 (18.6%)	582 (18.0%)	
TNM stage			<0.001			0.029
I	5885 (90.5%)	2830 (87.4%)		2787 (86.1%)	2830 (87.4%)	
II	599 (9.2%)	382 (11.8%)		437 (13.5%)	382 (11.8%)	
III	14 (0.2%)	24 (0.7%)		11 (0.3%)	24 (0.7%)	
IV	3 (0.0%)	2 (0.1%)		3 (0.1%)	2 (0.1%)	
Mortality						
All-cause	276 (4.2%)	77 (2.4%)	<0.001	151 (4.7%)	77 (2.4%)	<0.001
Total cancer	183 (2.8%)	57 (1.8%)	0.002	99 (3.1%)	57 (1.8%)	0.001
Thyroid cancer	52 (0.8%)	20 (0.6%)	0.388	30 (0.9%)	20 (0.6%)	0.201

a Propensity score matching was performed in a 1:1 ratio based on age at the operation, sex, tumor size, ETE, and LNM status.

b The VD group is the case group with oral vitamin D supplementation for at least 6 months.

c OP extent, operation extent.

d TT, total thyoridectomy.

e ETE, extrathyroidal extension, was confirmed by final pathologic results and included microscopic and gross ETE.

f LNM status, lymph node metastasis status, was defined as follows according to the final pathological results; i) ‘no LNM’ indicates no lymph node metastasis, ii) ‘central LNM’ indicates lymph node metastasis only in the central compartment, and iii) ‘lateral LNM’ indicates lateral compartment lymph node metastasis regardless of central compartment lymph node metastasis.

After PSM, 3,238 patients were assigned to each group. The mean ages of patients in the control and VD groups were 48.5 ± 11.1 and 48.6 ± 10.8 years, respectively (*p*=0.762). There were 320 (9.9%) male subjects in the control group and 342 (10.6%) in the VD group (*p*=0.389). The number of patients who underwent total thyroidectomy was 2,806 (88.5%) in control group and 3,092 (95.6%) in VD group (*p*<0.001). Tumor size was not different between the two groups (control, 1.2 ± 1.0 cm vs. VD group, 1.2 ± 0.9 cm, *p*=0.613). ETE was confirmed in 1,953 patients (60.3%) in the control group and in 1,963 patients (60.6%) in the VD group (*p*=0.819). Central and lateral LNM was confirmed in 789 (24.4%) and 602 (18.6%) patients in the control group and in 830 (25.6%) and 582 (18.0%) patients in the VD group, respectively (*p*=0.473). There were more patients with TNM stage I and III in the VD group and more patients with TNM stage II in the control group (p=0.029). The number of patients who died from any cause was 151 (4.7%) in the control group and 77 (2.4%) in the VD group (*p*<0.001). The number of patients who died from total cancer-related causes was 99 (3.1%) in the control group and 57 (1.8%) in the VD group (*p*=0.001). The number of patients who died from thyroid cancer-related causes did not differ between the two groups (control group, n=30 (0.9%) vs. VD group, n=20 (0.6%), *p*=0.201). After conducting PSM, a total of 151 and 77 participants in the control and VD groups, respectively, died during the study period, and there were no significant differences in the causes of death by organ system between the two groups. There were no significant differences in all-cause mortality, total cancer mortality, and thyroid cancer mortality between the two groups classified by extent of operation, with p-values of 0.116, 0.149, and 0.389, respectively. The all-cause mortality was 2.6% (n=146) in stage I, 9.0% (n=74) in stage II, 14.3% (n=5) in stage III, and 60.0% (n=3) in stage I. Regarding the mortality rates for all cancer types, the corresponding rates for stages I, II, III, and IV were 1.6% (n=91), 7.0% (n=57), 14.3% (n=5), and 60.0% (n=3), respectively. The thyroid cancer mortality rates also varied, with rates of 0.4% (n=22), 2.7% (n=22), 8.6% (n=3), and 60.0% (n=3) in stages I to IV, respectively.

### Comparisons of mortalities according to vitamin D supplementation

**Figure 2:**
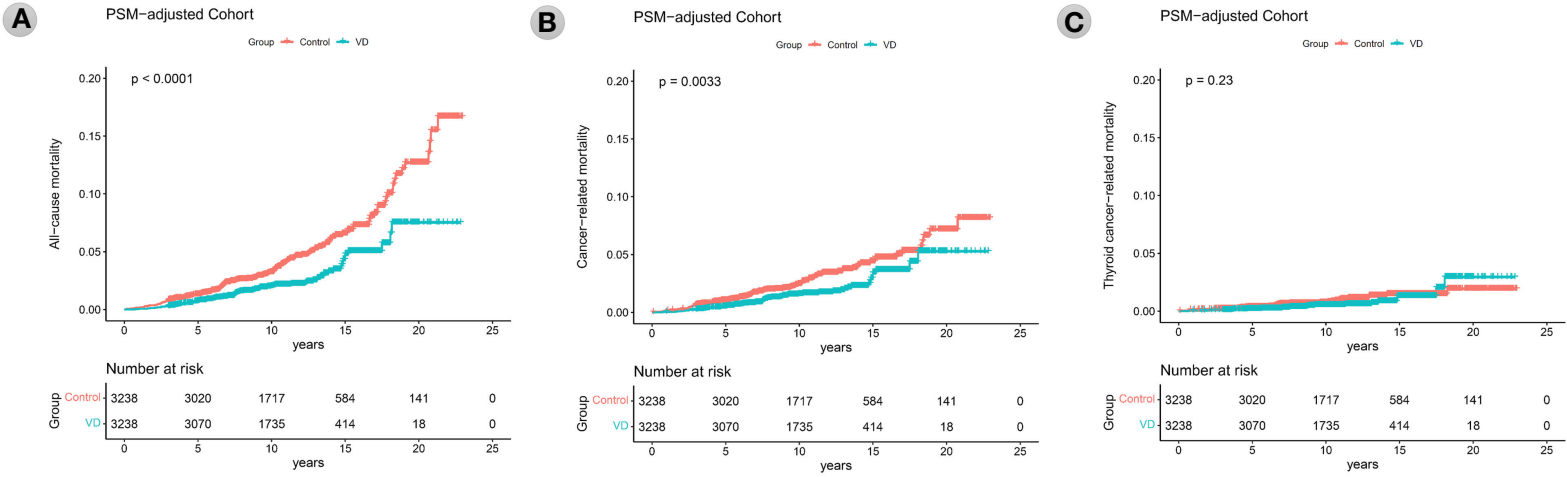
Kaplan–Meier survival curves of all-cause mortality **(A)**, total cancer mortality **(B)**, thyroid cancer mortality **(C)** according to the groups (vitamin D supplementation) in the propensity score matching-adjusted cohort. PSM, propensity score matching which was performed in a 1:1 ratio based on age at the operation, sex, tumor size, ETE, and LNM status. VD, VD group which is the case group with oral vitamin D supplementation for at least 6 months.

During a median follow-up of 10.7 ± 4.2 years, 228, 156, and 50 patients died from all-cause, total cancer-related, and thyroid cancer-related causes, respectively. The analyses to compare all-cause, total cancer, and thyroid cancer mortality according to vitamin D supplementation are described in **Figure 2**. Kaplan−Meier survival curves showed that the patients of the VD group had significantly better all-cause mortality than patients of the control group (*p*<0.001). At 5, 10, 15, and 20 years after surgery, the all-cause mortality rates of the control group patients were 1.3%, 3.2%, 6.6%, and 12.7%, respectively, and those of the VD group patients were 0.7%, 2.0%, 4.8%, and 7.5%, respectively. There was also significantly better total cancer mortality among VD group patients than control group patients (*p*=0.003) according to Kaplan−Meier curve analysis. The 5-, 10-, 15-, and 20-year total cancer mortality rates in the control group were 1.0%, 2.4%, 4.4%, and 7.1%, respectively, and those in the VD group were 0.5%, 1.6%, 3.4%, and 5.2%, respectively (*p*=0.003). However, the Kaplan−Meier curve showed no difference in thyroid cancer mortality between the two groups (*p*=0.23). The 5-, 10-, 15-, and 20-year thyroid cancer mortality rates in the control group were 0.3%, 0.7%, 1.5%, and 1.9%, respectively, and those in the VD group were 0.2%, 0.5%, 1.3%, and 2.9%, respectively (*p*=0.23).

### Comparative analysis of factors affecting mortality

**Figure 3:**
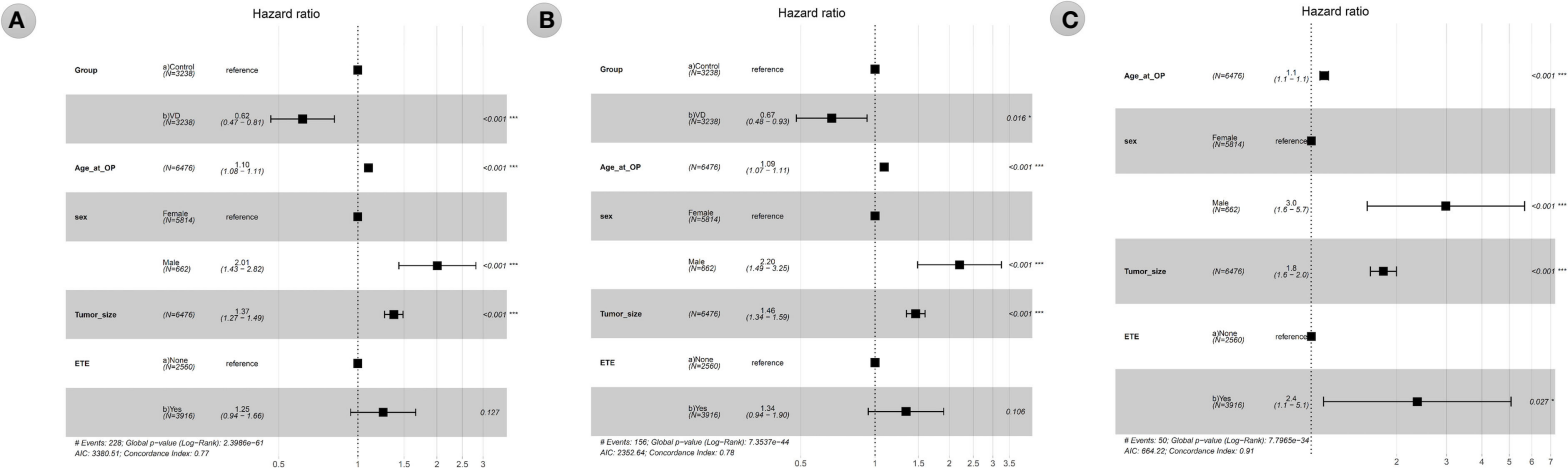
1 Forest plot of hazard ratios from multivariable Cox proportional hazards regression analysis of all-cause mortality **(A)**, total cancer mortality **(B)**, thyroid cancer mortality **(C)** according to the groups (vitamin D supplementation) in the propensity score matching-adjusted cohort. VD, VD group which is the case group with oral vitamin D supplementation for at least 6 months. Age_at_OP, Age at operation in years. ETE, extrathyroidal extension, was defined according to the final pathologic results and included microscopic and gross ETE.

The results of Cox proportional hazards regression analysis of the effect of vitamin D supplementation on all-cause, total cancer, and thyroid cancer mortality are summarized in **Table 2** and **Figure 3**. Cox analysis showed that vitamin D supplementation significantly reduced the hazard ratio (HR) for all-cause mortality to 0.572 (95% CI 0.443-0.739, *p*=0.001) and total cancer mortality to 0.627 (95% CI 0.464-0.847, *p*=0.016). However, thyroid cancer mortality was not affected by vitamin D supplementation in the Cox analysis. Cox analysis showed that the risk of all-cause mortality increased with age at surgery (HR 1.098, 95% CI 1.082-1.115, *p*<0.001), male sex (HR 2.009, 95% CI 1.433-2.815, *p*<0.001), and tumor size (HR 1.372, 95% CI 1.266-1.487, *p*<0.001). In Cox analysis, total cancer mortality was analyzed for increased risk with the same variables as all-cause mortality, including age at surgery (HR 1.09, 95% CI 1.071-1.109, *p*<0.001), male sex (HR 2.2, 95% CI 1.488-3.254, *p*<0.001), and tumor size (HR 1.462, 95% CI 1.34-1.594, *p*<0.001). According to Cox analysis, thyroid cancer mortality was increased in patients with ETE (HR 2.366, 95% CI 1.103-5.072, *p*=0.027) as well as in patients with older age at operation (HR 1.111, 95% CI 1.073-1.15, *p*<0.001), male sex (HR 2.983, 95% CI 1.573-5.656, *p*=0.001), and larger tumor size (HR 1.797, 95% CI 1.615-1.998, *p*<0.001).

**Table 2:** Cox proportional hazards regression analysis for all-cause mortality, total cancer mortality and thyroid cancer mortality in thyroid cancer patients in the propensity score matching-adjusted cohort.

	All-cause mortality		Total cancer mortality		Thyroid cancer mortality	
	HR, 95% CI^a^	*p*-value	HR, 95% CI^a^	*p*-value	HR, 95% CI^a^	*p*-value
Vitamin D supplementation	0.617, 0.467-0.814	0.001	0.668, 0.48-0.928	0.016	n/a^b^	n/a^b^
Age at operation (years)	1.098, 1.082-1.115	<0.001	1.09, 1.071-1.109	<0.001	1.111, 1.073-1.15	<0.001
Sex (Male)	2.009, 1.433-2.815	<0.001	2.2, 1.488-3.254	<0.001	2.983, 1.573-5.656	0.001
Tumor size (cm)	1.372, 1.266-1.487	<0.001	1.462, 1.34-1.594	<0.001	1.797, 1.615-1.998	<0.001
ETE^c^	n/a^b^	n/a^b^	n/a^b^	n/a^b^	2.366, 1.103-5.072	0.027

a HR, 95% CI, hazard ratio, 95% confidential interval.

b n/a, not applicable.

c ETE, extrathyroidal extension, was defined according to the final pathologic results and included microscopic and gross ETE.

## Discussion

This retrospective cohort study included 9,739 DTC patients with available information on vitamin D supplementation. We analyzed mortalities in the context of three categories based on cause of death, providing differentiated information on the effects of vitamin D supplementation on various causes of death. We found that vitamin D supplementation was associated with reduced all-cause and total cancer mortality in patients with DTC. These results suggest that vitamin D supplementation could be a potential prognostic factor in DTC patients. To our knowledge, this is the first study to analyze the effect of vitamin D supplementation on mortality in patients with thyroid cancer. To avoid selection bias, we performed PSM analysis to minimize the influence of potential confounders, including established prognostic factors (age, sex, tumor size, ETE, and LNM) (3, 5). After PSM, 6,476 patients with a median follow-up of 10.7 years were analyzed to evaluate the effect of vitamin D supplementation on mortality in DTC patients. Therefore, the present study with a large sample size and long-term follow-up provides reliable information.

Vitamin D can act in multiple carcinogenesis stages, reduce the invasiveness and metastasis of tumors, and modulate the immune system (12). Francesca cialdai et al. reported potential antineoplastic functions of vitamin D (8). Cell cycle arrest, apoptosis, epithelial-mesenchymal transition, epithelial-mesenchymal transition, CD8+ T-cell infiltration, and anti-inflammation are potential antitumorigenic mechanisms of vitamin D action. Vitamin D regulates multiple signaling pathways through activation of vitamin D receptor (VDR) transcription factor. Phosphoinositide 3-kinase and mitogen-activated protein kinase downstream of ligand-bound VDR can be activated and modulate microRNA expression and cancer stem cell biology. The VDR-retinoid X receptor complex and vitamin D elements in target genes alter gene transcription in response to vitamin D. However, the role of vitamin D in carcinogenesis is still under debate.

The beneficial effect of vitamin D supplementation on cancer mortality was reported in a meta-analysis of 10 randomized controlled trials (summary RR, 0.87; 95% CI, 0.79-0.96) (12). A recent randomized controlled trial (VITAL) also reported that vitamin D supplementation reduced the HR for total cancer mortality to 0.79 and 0.75 at time points other than the first-year and second-year follow-ups (11). However, another randomized controlled trial (D-Health trial) including 21,315 older participants (≥60 years) did not show the beneficial effect of vitamin D supplementation for the reduction of all-cause mortality (19). Our study revealed that vitamin D supplementation reduces all-cause and total cancer mortality and supports the efficacy of vitamin D supplementation, albeit to varying degrees.

However, we could not observe a significant improvement in thyroid cancer mortality in thyroid cancer patients who had vitamin D supplementation. Because DTC patients have excellent clinical outcomes, with a 10-year cancer-specific survival of 97.2%, only 50 patients of 6,476 patients died from thyroid cancer during the follow-up period of 10.7 years (20). The nonsignificant result might be attributed to our study being of short a duration to observe sufficient mortality events, considering the very low mortality rate of thyroid cancer.

The VITAL study suggested that the effect of vitamin D intake in reducing metastatic and advanced cancers is due to a general mechanism affecting the body, not a site-specific mechanism (11). The insignificant effects of vitamin D supplementation on the reduction in the risk of thyroid cancer mortality may be due to the lack of a site-specific effect of vitamin D supplementation. Therefore, further studies will be needed to ascertain the effect of vitamin D supplementation with large numbers of patients and long-term follow-up periods, including sufficient numbers of thyroid cancer-specific deaths.

Thyroid cancer was reported to be associated with low levels of vitamin D from the perspective of clinical or pathological aggressiveness *in vitro*, case-control, and cohort studies, and the results varied from study to study (3). Stepien et al. reported that vitamin D levels were lower in thyroid cancer patients than in normal subjects, and Kim et al. reported that patients with lower vitamin D levels were associated with more aggressive features of thyroid cancer (14, 15). However, there were also previous studies that did not show a significant association between vitamin D levels and aggressive characteristics of cancer in thyroid cancer patients (16, 21, 22).

A previous multicenter cohort study showed that older age at diagnosis, male sex, larger tumor size over 2 cm, presence of ETE, lateral cervical LNM, distant metastasis, and TNM stages at diagnosis were independent risk factors for disease-specific mortality in DTC patients (23). Our results suggest that male sex is most likely to be associated with an increased risk of all-cause, total cancer, and thyroid cancer mortality (HR 2.009, HR 2.2, and HR 2.983, respectively). In addition to male sex, large tumor size and old age at time of operation were determined to have a significant positive correlation with increases in all-cause, total cancer, and thyroid cancer mortality. Sex, tumor size, age, and ETE are well-known prognostic factors associated with the aggressiveness and prognosis of thyroid cancer (3, 5). In this study, these factors were adjusted using PSM to confirm an effect of vitamin D on thyroid cancer mortality.

This study has some limitations. First, as this study is a retrospective observational cohort analysis, it was not possible to completely eliminate the potential for selection bias, and the detailed information could not be comprehensively reviewed. Second, we could not analyze the mechanism by which vitamin D positively affects mortality, and the criteria for vitamin D supplementation were not uniform. Third, since the vitamin D level before and after vitamin D supplementation was not included in the analysis, it was not possible to analyze the effect of the vitamin D level or its increase or decrease on the prognosis of thyroid cancer. Fourth, other possible confounding factors, such as socioeconomic status, comorbidity, and lifestyle, were not analyzed. In the future, well-designed studies are needed to analyze the relationship between the prognosis of thyroid cancer patients and vitamin D supplementation regimens.

## Conclusion

Vitamin D supplementation was positively associated with all-cause and total cancer mortality in patients with DTC. We suggest that vitamin D supplementation has potential to be a modifiable prognostic predictor regarding reduced mortality in patients with DTC. Further research will be needed to clarify the effect of vitamin D supplementation on DTC.

## Data availability statement

The raw data supporting the conclusions of this article will be made available by the corresponding authors upon request.

## Ethics statement

The studies involving human participants were reviewed and approved by Seoul National University Hospital. Written informed consent for participation was not required for this study in accordance with the national legislation and the institutional requirements.

## Author contributions

(I) Conception and design: SJK, HSC, and YJP. (II) Administrative support: SJK and YJP. (III) Provision of study materials or patients: SJK, SWC, KEL, DJP, and YJP. (IV) Collection and assembly of data: JHA, and HSC. (V) Data analysis and interpretation: JHA, and HSC. (VI) Manuscript writing: all authors. All authors contributed to the article and approved the submitted version.
