# Supplementary material for: The association between vitamin D supplementation and the long-term prognosis of differentiated thyroid cancer patients: a retrospective observational cohort study with propensity score matching
Source: Front Endocrinol (Lausanne). 2023 Jun 13;14:1163671. doi: 10.3389/fendo.2023.1163671 (PMC10296193; doi:10.3389/fendo.2023.1163671)
Supplement: Supplementary file 1 [file DataSheet_1.docx]

**Supplementary Table 1. Comparison of differences between the group taking vitamin D and the control group according to causes of death in the original cohort and the propensity score matching cohort.**

|  | Original unmatched cohort | | | PSM-adjusted cohort^a^ | | |
| --- | --- | --- | --- | --- | --- | --- |
|  | Control group  (n=276) | VD group^b^  (n=77) | *p*-value | Control group  (n=151) | VD group^b^  (n=77) | *p*-value |
| Thyroid cancer | 52 (18.8%) | 20 (26.0%) | 0.170 | 30 (19.9%) | 20 (26.0%) | 0.292 |
| Other cancer | 134 (48.6%) | 40 (51.9%) | 0.598 | 71 (47.0%) | 40 (51.9%) | 0.481 |
| Adrenal cancer | 1 (0.7%) | 1 (2.5%) |  | 1 (1.4%) | 1 (2.5%) |  |
| Bone and Cartilage cancer | 1 (0.7%) | 0 (0.0%) |  | 1 (1.4%) | 0 (0.0%) |  |
| Brain cancer | 3 (2.2%) | 1 (2.5%) |  | 2 (2.8%) | 1 (2.5%) |  |
| Breast cancer | 15 (11.2%) | 10 (25.0%) |  | 8 (11.3%) | 10 (25.0%) |  |
| Female genital tract cancer | 5 (3.7%) | 2 (5.0%) |  | 5 (7.0%) | 2 (5.0%) |  |
| Gastrointestinal tract cancer | 24 (17.9%) | 5 (12.5%) |  | 12 (16.9%) | 5 (12.5%) |  |
| Genitourinary tract cancer | 5 (3.7%) | 1 (2.5%) |  | 5 (7.0%) | 1 (2.5%) |  |
| Hematologic disease and Lymphoma | 13 (9.7%) | 7 (17.5%) |  | 7 (9.9%) | 7 (17.5%) |  |
| Hepatobiliary cancer | 29 (21.6%) | 6 (15.0%) |  | 10 (14.1%) | 6 (15.0%) |  |
| Meningioma | 1 (0.7%) | 1 (2.5%) |  | 0 (0.0%) | 1 (2.5%) |  |
| Nasopharyngeal cancer | 7 (5.2%) | 0 (0.0%) |  | 4 (5.6%) | 0 (0.0%) |  |
| Prostate cancer | 1 (0.7%) | 0 (0.0%) |  | 1 (1.4%) | 0 (0.0%) |  |
| Respiratory tract cancer | 24 (17.9%) | 5 (12.5%) |  | 12 (16.9%) | 5 (12.5%) |  |
| Skin cancer | 2 (1.5%) | 1 (2.5%) |  | 2 (2.8%) | 1 (2.5%) |  |
| Unstated cancer | 3 (2.2%) | 0 (0.0%) |  | 1 (1.4%) | 0 (0.0%) |  |
| Other benign disease | 90 (32.6%) | 17 (22.1%) | 0.075 | 50 (33.1%) | 17 (22.1%) | 0.084 |

^a^ Propensity score matching was performed in a 1:1 ratio based on age at the operation, sex, tumor size, extrathyroidal extension, and lymph node metastasis status.

^b^ The VD group is the case group with oral vitamin D supplementation for at least 6 months.

**Supplementary Table 2. Basic characteristics between two groups by surgical extent in the original unmatched cohort and the propensity score matched cohort.**

|  | Original unmatched cohort | | | PSM-adjusted cohort^a^ | | |
| --- | --- | --- | --- | --- | --- | --- |
|  | Less than TT^b^  (n=940) | TT^b^  (n=8,671) | *p*-value | Less than TT^b^  (n=507) | TT^b^  (n=5,898) | *p*-value |
| Group |  |  | <0.001 |  |  | <0.001 |
| Control | 796 (84.7%) | 5579 (64.3%) |  | 363 (71.6%) | 2806 (47.6%) |  |
| VD^c^ | 144 (15.3%) | 3092 (35.7%) |  | 144 (28.4%) | 3092 (52.4%) |  |
| Age at operation (years) | 45.5±11.3 | 47.1±11.3 | <0.001 | 47.0±10.9 | 48.7±10.9 | 0.001 |
| Sex |  |  | 0.295 |  |  | 0.310 |
| Female | 778 (82.8%) | 7296 (84.1%) |  | 462 (91.1%) | 5284 (89.6%) |  |
| Male | 162 (17.2%) | 1375 (15.9%) |  | 45 (8.9%) | 614 (10.4%) |  |
| Tumor size (cm) | 1.3±1.3 | 1.2±0.9 | <0.001 | 1.3±1.3 | 1.2±0.9 | 0.008 |
| ETE^d^ |  |  | <0.001 |  |  | <0.001 |
| No | 640 (68.1%) | 3531 (40.7%) |  | 326 (64.3%) | 2203 (37.4%) |  |
| Yes | 300 (31.9%) | 5140 (59.3%) |  | 181 (35.7%) | 3695 (62.6%) |  |
| LNM^e^ status |  |  | <0.001 |  |  | <0.001 |
| No LNM^e^ | 798 (84.9%) | 4966 (57.3%) |  | 412 (81.3%) | 3220 (54.6%) |  |
| Central LNM^e^ | 82 (8.7%) | 2108 (24.3%) |  | 56 (11.0%) | 1560 (26.4%) |  |
| Lateral LNM^e^ | 60 (6.4%) | 1597 (18.4%) |  | 39 (7.7%) | 1118 (19.0%) |  |
| TNM stage |  |  | <0.001 |  |  | <0.001 |
| I | 902 (96.0%) | 7702 (88.8%) |  | 479 (94.5%) | 5079 (86.1%) |  |
| II | 37 (3.9%) | 927 (10.7%) |  | 27 (5.3%) | 780 (13.2%) |  |
| III | 1 (0.1%) | 37 (0.4%) |  | 1 (0.2%) | 34 (0.6%) |  |
| IV | 0 (0.0%) | 5 (0.1%) |  | 0 (0.0%) | 5 (0.1%) |  |
| Mortality |  |  |  |  |  |  |
| All-cause | 39 (4.1%) | 296 (3.4%) | 0.283 | 24 (4.7%) | 195 (3.3%) | 0.116 |
| Total cancer | 23 (2.4%) | 202 (2.3%) | 0.911 | 17 (3.4%) | 132 (2.2%) | 0.149 |
| Thyroid cancer | 7 (0.7%) | 62 (0.7%) | 1.000 | 6 (1.2%) | 43 (0.7%) | 0.389 |

^a^ Propensity score matching was performed in a 1:1 ratio based on age at the operation, sex, tumor size, ETE, and LNM status.

^b^ Total thyroidectomy.

^c^ The VD group is the case group with oral vitamin D supplementation for at least 6 months.

^d^ ETE, extrathyroidal extension, was confirmed by final pathologic results and included microscopic and gross ETE.

^e^ LNM status, lymph node metastasis status, was defined as follows according to the final pathological results; i) ‘no LNM’ indicates no lymph node metastasis, ii) ‘central LNM’ indicates lymph node metastasis only in the central compartment, and iii) ‘lateral LNM’ indicates lateral compartment lymph node metastasis regardless of central compartment lymph node metastasis.
